# Research on Environmental Influencing Factors of Overweight and Obesity in Children and Adolescents in China

**DOI:** 10.3390/nu14010035

**Published:** 2021-12-23

**Authors:** Yaru Guo, Xiaojian Yin, Yi Sun, Ting Zhang, Ming Li, Feng Zhang, Yuan Liu, Jianyi Xu, Dandan Pei, Tianlong Huang

**Affiliations:** 1 Key Laboratory of Adolescent Health Assessment and Exercise Intervention, Ministry of Education, College of Physical Education & Health, East China Normal University, Shanghai 200241, China; 17321210960@163.com (Y.G.); sunyi0084@163.com (Y.S.); noway1982@163.com (T.Z.); liming23416@163.com (M.L.); fzhang1988@126.com (F.Z.); yliu0809@163.com (Y.L.); kavinyee@163.com (J.X.); pei18621055443@163.com (D.P.); HTLONG192@163.com (T.H.); 2 College of Economics and Management, Shanghai Institute of Technology, Shanghai 201418, China

**Keywords:** children and adolescents, overweight and obesity, environment, influencing factors

## Abstract

This study aimed to explore the impact of environmental factors such as latitude, altitude, family socioeconomic status (SES), and level of urbanization on overweight and obesity (ow/ob) in children and adolescents. The participants comprised 26,120 children and adolescents aged 10–18 from 16 provinces in China. Differences in the prevalence of ow/ob under different environmental conditions were evaluated by the chi-square test. The influence of various environmental factors on ow/ob was obtained by logistic regression analysis. We found that (1) the prevalence of ow/ob fell between from 19.2% to 11.9% at 10 years old and from 13.8% to 6% at 18 years old; (2) latitude, family SES, income, and urbanization level are positively correlated with the prevalence of ow/ob; and (3) altitude has a negative correlation with the prevalence of ow/ob. The prevalence of ow/ob decreased with age in children and adolescents aged 10–18, and the risk of ow/ob showed significant differences in latitude, altitude, family SES level, gross domestic product (GDP), and level of urbanization.

## 1. Introduction

In recent decades, the prevalence of overweight and obesity has risen rapidly around the world [1], becoming a major public health concern in various fields internationally. In China, the rapid development of the economy and lifestyle changes both resulted in a rapid increase in overweight and obesity rates among children and adolescents [2]. The occurrence of overweight and obesity has a serious negative impact on the physical and mental health of children and adolescents. Studies have shown that overweight and obese people aged 15 to 18 have a much higher risk of hypertension than those of normal weight [3]. Moreover, most obese adolescents show varying degrees of decreased self-esteem, accompanied by mental health problems (such as anxiety, tension, and loneliness) that are highly related to high-risk behaviors [4,5]. What compounds this issue is the fact that nearly 80% of obese adolescents are still obese in adulthood [6]. At this time, obesity and other diseases in adulthood affect each other and reduce life expectancy [7]. Based on this, analyzing the causes of overweight and obesity in children and adolescents in China will be of great significance to effectively prevent and control their occurrence.

The susceptibility of children and adolescents to obesity is determined by genetic factors, and whether obesity occurs is mainly determined by environmental factors [8,9,10,11]. In terms of natural environmental factors, China has a vast territory, complex terrain, and climate. Due to differences in altitude, temperature, humidity, and light conditions in various regions, there are also differences in food composition and intake of various nutrients. To adapt to the natural environment, Chinese children and adolescents have changed their body composition and living habits accordingly. The main distribution characteristics of overweight and obesity in children and adolescents are as follows: their prevalence is highest in north China and northeast China, and lower in southwest and south China [12]. At the same time, under the influence of factors such as altitude, sunshine time, and temperature, the vital capacity (the greatest volume of air that can be expelled from the lungs after taking the deepest possible breath) of adolescent boys also shows a gradual decrease from northeast to west and south [13]. Wang [14] pointed out in his research that the main characteristics of human height are as follows: people located in high latitudes, low altitudes, pastoral areas, and economically developed areas are taller, while people located in low latitudes, high altitudes, agricultural areas, and economically relatively underdeveloped areas are shorter. It can be seen that natural environmental factors have an important impact on people’s physical growth and development.

While overweight and obesity in children and adolescents are affected by natural environmental factors, they are still affected by behavioral factors such as eating habits, physical activity status, and lifestyle. Cohen et al. [15] believe that the behaviors of children and adolescents have not yet formed into habits like those of adults, and their plasticity is greater. Their behaviors are more easily affected by the surrounding social environment, and the decisive factor of the social environment is family socioeconomic status (SES). Family SES can be used as a comprehensive reflection of parents’ educational level, family income level, and occupation. Due to the different stages of economic development in different countries, the results of SES on overweight and obesity are not consistent. In developed countries such as the United States, the United Kingdom, and Germany, children in families with lower SES are at higher risk of obesity [16,17,18]; in contrast to the findings of developing countries such as India, Vietnam, and Nigeria, students with higher socioeconomic levels are overweight and the obesity rate is higher [18]. In China, in general, the higher the annual family income, the higher the rate of overweight and obesity in children and adolescents; the higher the education level of the parents, the lower the rate of overweight and obesity. It can be seen that with the development of the social economy, social environmental factors have gradually become an important factor leading to overweight and obesity in children and adolescents.

Based on the above background, this study uses a comparative analysis of children and adolescents at different altitudes and latitudes to understand the distribution characteristics and prevalence of overweight and obesity in children and adolescents aged 10 to 18 in China. At the same time, from the perspective of natural environmental factors and social environmental factors, the impact of environmental factors such as latitude, altitude, family SES, and level of urbanization on overweight and obesity in children and adolescents is discussed, to improve the physical and healthy development of children and adolescents, and develop targeted control of children. Individualized interventions for overweight and obesity in adolescents provide a relevant theoretical basis.

## 2. Materials and Methods

### 2.1. Data Source and Participants

The data are from test data of “development of new assessment methods and evaluation standards for the physical health of Chinese children and adolescents” which is a major project of the key laboratory of the Ministry of Education on “Health Assessment and Exercise Intervention of Adolescents”. Ethical approval was obtained from East China Normal University’s Human Experiment Ethics Committee. Grant N: HR2016/12055.

In October 2016, researchers surveyed the basic information and family status of nearly 100,000 children and adolescents aged 7 to 18 in China through the “Children and Adolescents’ Family Situation Survey Form”. This study adopted the “stratified cluster random sampling” method to randomly select 16 provinces in Shanghai, Heilongjiang, Hebei, Henan, Shanxi, Jiangsu, Zhejiang, Anhui, Jiangxi, Sichuan, Guizhou, Fujian, Hainan, Xinjiang, Jilin, and Yunnan. A total of 26,120 students, comprising children and adolescents aged 10–18, formed the research objects of this study.

### 2.2. Geographical Division of Research Objects

Based on information of the province, city, and district/town where the research object is located, the specific latitude and altitude of the research object can be found on the website of the Ministry of Natural Resources [19] and the altitude query website [20]. They are divided as follows.

The division of latitude area: In this study, the survey scope is wide, and the latitude span of the research objects is large. The lowest latitude is located in Sanya, Hainan (18°25′ N), and the highest latitude is located in Daxing’anling (50°42′ N) of Heilongjiang, north and south. The span is about 32°. In geography, 30–40° N is usually taken as the mid-latitude zone of China’s mainland [21,22], and the natural environment of China above 40° N and 30–40° N is quite different; the natural environment below 30° N is the same [23]. Based on the above research, in the absence of general classification, this research divides the latitude of the research object into the following three regions for comparative research: low latitude (latitude < 30° N), middle latitude (30° N ≤ latitude ≤ 40° N), high latitude (latitude > 40° N).

The division of altitude regions: In this study, the lowest altitude of the research objects is located in Shanghai (2.8 m), and the highest altitude is located in Kunming, Yunnan (1891.4 m). The altitude of the research object is quite different. However, current research lacks a unified division of altitude. Therefore, in this study, the altitude of the research object is divided as follows: low altitude (altitude < 500 m), medium altitude (500 m ≤ Altitude ≤ 1000 m), high altitude (altitude > 1000 m).

### 2.3. Anthropometry and the Classification Criteria for Overweight and Obesity

Body height was determined using a mechanical height gauge and measured, without shoes, to the nearest 0.1 cm. Bodyweight was measured in light clothing, without shoes, to the nearest 0.1 kg using an electronic scale.

We calculated the body mass index (BMI) = weight (kg)/height^2^ (m^2^) of the subject using height and weight measurements. The classification of overweight and obesity in children and adolescents is based on the BMI screening criteria for overweight and obesity among school-age children and adolescents aged 5–19 years proposed by the World Health Organization (WHO) in 2007 [24].

### 2.4. Mathematical Processing

This study uses indicators such as parents’ educational level, occupation, and family income as the criteria for measuring SES in adolescent families. We also included three socio-economic indicators, namely, gross domestic product (GDP) per capita, Engel coefficient, and urbanization level. These indicators come from the statistical yearbooks of the National and Provincial Bureau of Statistics of China [25]. Among them, SES reflects personal economic conditions, while others reflect the overall economy.

#### 2.4.1. Socioeconomic Status

Family SES calculation is as follows [26]: (1) Assignment: education level is scored according to the number of years of education; scores for all occupations are between 16 and 90 points [27]; family monthly income is scored 2 points as “less than RMB 2000”, “RMB 2001–5000” is worth 5 points, “RMB 5001–8000” is worth 8 points, and “RMB 8000 or more” is worth 10 points; (2) screening or conversion; (3) dealing with missing values; (4) calculation: after using SPSS 25.0.0 to convert the above three variables into standard scores, principal component analysis is performed to obtain SES. The SES score is directly proportional to the socioeconomic status of the family. The total score of the subjects is between −2.58 and 1.75, with an average value of 0.00. According to the SES score, this paper divides the family’s SES into three categories: low, middle, and high.

#### 2.4.2. Gross Domestic Product

GDP per capita classification criteria are as follows [28]: low-income economies (less than RMB 6679); lower-middle-income economies (RMB 6680–26,261); middle- and upper-income economies (RMB 26,262–81,240); high-income economy (above RMB 81,240).

#### 2.4.3. Engel Coefficient

Engel coefficient classification standard is as follows [29]: poverty (Engel coefficient, >59%; adequate (Engel coefficient, 50–59%); well-off (Engel coefficient, 40–50%); rich (Engel coefficient, 30–40%); the richest (Engel coefficient, <30%).

#### 2.4.4. Urbanization Level

Urbanization rate classification standard is as follows: first level (lowest degree of urbanization, ≤40.00%); second level (lower degree of urbanization, 40.01–50.00%); third level (middle and upper degree of urbanization, 50.01–60.00%); fourth level (highest degree of urbanization, >60.00%).

### 2.5. Statistical Analysis

First, SPSS 25.0.0 software was used to carry out the conversion of index standards and principal component analysis, and the household SES was calculated according to the three indexes of education level, occupation, and family monthly income. Secondly, we calculated the height, weight, and BMI of the research objects under different environmental conditions, expressed as the mean ± standard deviation, and used the chi-square test to detect overweight and obesity in children and adolescents under different natural and social environmental conditions. For comparison, when *p* < 0.05, it was statistically significant. Finally, through logistic regression analysis, we explored the impact of natural and social environmental factors on overweight and obesity. We used Excel 2019 for chart production.

## 3. Results

### 3.1. Basic Situation

The distribution characteristics of the 26,120 students are shown in Table 1.

#### 3.1.1. Distribution Characteristics of Nutritional Status of Children and Adolescents Aged 10–18

**Figure 1:**
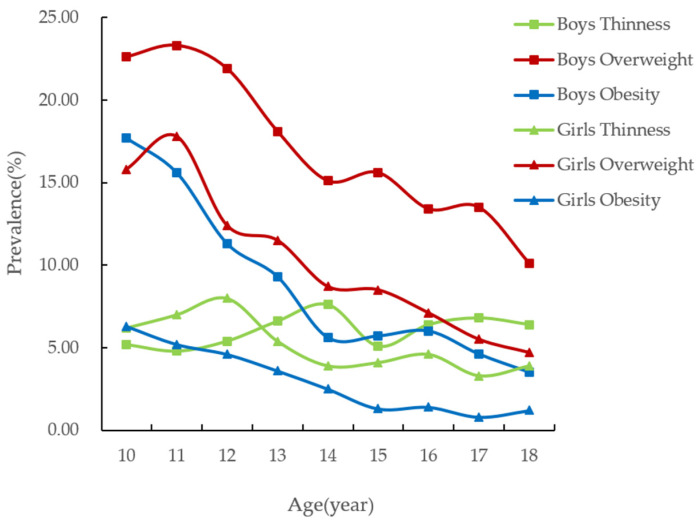
Prevalence of different nutritional status in children and adolescents aged 10–18 years (%).

From Table 2 and Figure 1, it can be seen that, in general, the overweight and obesity rates of male and female students generally show a decreasing trend with age, and the overweight and obesity rates of boys are higher than those of girls; the rate of thinness among boys has not changed much. However, the prevalence of thinness among girls decreases year by year with age. The prevalence of overweight and obesity in boys decreased from 22.6% and 17.7% at the age of 10 to 10.1% and 3.5% at the age of 18, respectively; the prevalence of overweight and obesity in girls decreased from 15.8% and 6.3% at the age of 10 to 4.7% and 1.2% at 18, respectively. However, in terms of overweight, both male and female students peaked at the age of 11, at 23.3% and 17.8%, respectively.

#### 3.1.2. Distribution Characteristics of Nutritional Status of Children and Adolescents in Various Provinces

It can be seen from Table 3 that most of the high thinness prevalence is concentrated in coastal areas. Among them, the three provinces with the highest thinness prevalence for males and females are Jilin 9.3%, Jiangxi 9.0%, Hainan 8.7%, Jiangxi 9.8%, and Anhui 9.1%, Jilin 7.3%; male and female low thinness prevalence in Shanghai, Jiangsu, Xinjiang and Shanghai, Sichuan, and Jiangsu provinces are 2.6%, 3.5%, 3.8% and 2.6%, 2.7%, and 3.3%, respectively. Male and female overweight and obesity prevalence are higher in high-latitude regions. For example, the provinces with the highest overweight prevalence for males and females are Fujian 23.5%, Shanghai 21.8%, Heilongjiang 20.7%, Heilongjiang 13.9% and Shanghai 13.6%, Xinjiang 13.2%; the provinces with low male and female overweight prevalence are Jiangxi, Guizhou, Hainan and Jiangxi, Guizhou, Henan, and their overweight prevalence is 10.6%, 13.0%, 13.3%, and 4.2%, 6.7%, 8.0%, respectively. The obesity prevalence for boys and girls is the highest in Heilongjiang (13.9% and 5.0%, respectively), followed by Jilin (11.9% and 4.3%, respectively); the provinces with lower obesity prevalence for boys and girls are Jiangxi and Jiangsu, Yunnan and Guizhou, and Jiangxi and Sichuan, respectively.

### 3.2. Research on Natural Environmental Factors Affecting Nutritional Status of Children and Adolescents

It can be seen from Table 4 that children and adolescents located at different latitudes and altitudes have significant differences in the prevalence of different nutritional statuses (*p* < 0.05). In terms of latitude, the prevalence of male and female thinness was the lowest at mid-latitudes of 5.2% and 4.4%, respectively. The prevalence of thinness in low-latitude and high-latitude regions was not much different; the prevalence of overweight and obesity for males and females was similar. The increase in latitude increases, from 15.5%, 7.3% and 8.8%, 2.6% at low latitudes to 19.9%, 12.2% and 13.4%, 3.9% at high latitudes. In terms of altitude, the prevalence of thinness in men and girls was the lowest at medium altitudes (5.1% and 3.7%, respectively), and the highest at low altitudes (6.4% and 5.7%, respectively); the prevalence of overweight and obesity in men and girls was highest at medium altitude (18.7%, 10.8% and 13%, 3.1%, respectively) and lowest at high altitude (13.8%, 5.9% and 8.7%, 1.8%, respectively).

### 3.3. Research on Social Environmental Factors Affecting Nutritional Status of Children and Adolescents

It can be seen from Table 5 that the prevalence of different nutritional statuses of children and adolescents in different households with SES level, urbanization degree, GDP, and Engel coefficient are significantly different (*p* < 0.05). In terms of family SES, overall, the prevalence of thinness in boys and girls decreased with the increase in SES, from 6.5% in low SES to 5.3% in high SES, but the prevalence of thinness in girls was higher in SES. The prevalence of overweight and obesity in boys and girls increased with the increase of family SES, from 11.2%, 5.8%, 7.4%, and 2.9% at low SES to 19.8%, 10.3%, 12%, and 3% at high SES, respectively. In terms of the degree of urbanization, the prevalence of male and female thinness was the lowest when the degree of urbanization was the highest, 5.1% and 4.3%, respectively. The prevalence of the other three levels of urbanization was not much different; males and females were overweight. The prevalence rate increases with the increase in the degree of urbanization, from 15.6% and 9% at the lowest level of urbanization to 18% and 10.7% at the highest level of urbanization. On the whole, obesity prevalence is linked to the level of urbanization. In terms of GDP, the rate of thinness detection for boys and girls decreases with the increase in GDP and is highest when GDP is low (6.4%). In general, it decreases from 7.5% for low-middle income to 5% for high income; overall, the prevalence of overweight and obesity is the lowest in the low-middle income group, 8.6% and 4.1%, respectively. The prevalence of obesity in the middle and upper-income groups is 6.2%, and the prevalence of obesity in the high-income group is 14.6%. Generally speaking, the lower the Engel coefficient, the richer the people, the lower the rate of thinness detection (from 7.1% in the well-off period to 5.4% in the richest period), and the higher the prevalence of overweight and obesity (from 8.8% and 4% in the well-off period to 14.8% and 6.4% in the richest period, respectively). 

### 3.4. Logistic Regression Analysis of Influencing Factors of Overweight and Obesity in Children and Adolescents

#### 3.4.1. Logistic Regression Analysis of Influencing Factors of Overweight in Children and Adolescents

From Table 6, it can be seen that boys, low latitudes, low altitudes, low SES families, minimum urbanization, moderately low-income, and well-off families are used as references. The overweight prevalence of girls is lower than that of boys (OR = 0.55, 95%CI: 0.52–0.6), which has a very significant statistical significance in reducing the risk of overweight (*p* < 0.001). The prevalence of overweight in high-altitude areas was lower than that in low-altitude areas (OR = 0.79, 95%CI: 0.66–0.95), which had statistical significance in reducing the risk of overweight (*p* < 0.05). The prevalence of overweight in high-latitude areas is higher than that in low-latitude areas (OR = 1.33, 95%CI: 1.15–1.54), middle SES families (OR = 1.17, 95%CI: 1.03–1.34), and high SES families (OR= 1.41, 95%CI: 1.24–1.61) The prevalence of overweight is higher than that of families with low SES, middle and upper income (OR = 1.5, 95%CI: 1.02–2.21), and high income (OR = 1.66, 95%CI: 1.11–2.47) The prevalence of overweight is higher than that of low-middle income. Among them, families with high latitude and high SES have a very high statistical significance in increasing the risk of overweight (*p* < 0.001). Middle-SES families, middle-upper income, and high-income have statistical significance in increasing the risk of overweight (*p* < 0.05). However, the relationship between the degree of urbanization, Engel coefficient, and overweight in children and adolescents was not significant (*p* > 0.05).

#### 3.4.2. Logistic Regression Analysis of Factors Affecting Obesity in Children and Adolescents

It can be seen from Table 7 that boys, low latitudes, low altitudes, low SES families, minimum urbanization, moderately low-income, and well-off families are used as references. The obesity prevalence of girls is much lower than that of boys (OR = 0.31, 95%CI: 0.28–0.35), and the obesity prevalence in high-altitude areas is lower than that in low-altitude areas (OR = 0.61, 95%CI: 0.45–0.8). The reduction in the risk of obesity has a very high statistical significance (*p <* 0.001). The obesity prevalence in high-latitude regions is higher than that in low-latitude regions (OR = 1.52, 95%CI: 1.24–1.86), and the obesity prevalence when the degree of urbanization is highest is higher than when the degree of urbanization is lowest (OR = 1.29, 95%CI: 1.01–1.65), which is a risk factor for obesity. Among them, high latitude has a very significant statistical significance in increasing the risk of obesity (*p* < 0.001). The high degree of urbanization has a statistically significant increase in the risk of obesity (*p* < 0.05). The relationship between family SES, GDP, and Engel coefficient and obesity in children and adolescents was not significant (*p* > 0.05).

## 4. Discussion

### 4.1. Analysis of Overweight and Obesity Status in Children and Adolescents in China

The results of the study showed that the overall overweight and obesity rate of boys and girls showed a decreasing trend with age, and the overweight and obesity rates of boys were higher than those of girls. There are large differences in the prevalence of overweight and obesity among students in various provinces, cities, and autonomous regions in China. The prevalence of overweight and obesity in Heilongjiang, Shanghai, and other places is relatively high, while the prevalence of overweight and obesity in Guizhou and Yunnan is relatively low.

Many studies have shown that the prevalence of overweight and obesity among school-age children and adolescents is higher than that of adolescent children and adolescents [30,31]. The reason for this phenomenon may be related to the physical development characteristics of children and adolescents. Puberty is the stage from the onset of youth to the basic maturity of growth, and the age range is 10 to 19 years [32]. Children and adolescents enter puberty at around the age of 10 when their growth accelerates rapidly. On the other hand, the reason could be that the high prevalence of overweight and obesity among school-age children is related to poor self-control, parents’ fear of overfeeding because of insufficient food, and lack of exercise. The decline in the prevalence in adolescence may be due to the increase in age, the increase in health awareness of oneself and parents, the increase in body-shape requirements, and the strengthening of exercise and diet control [33].

Regarding the gender difference in the prevalence of overweight and obesity, the reason that the prevalence of overweight and obesity in boys is higher than that in girls may be due to the following: First, the tendency of Chinese parents to recognize the physical development of boys and girls will lead to the feeding of boys and girls. The difference is mainly manifested in the overfeeding of boys, which makes the prevalence of overweight and obesity higher than that of girls [34,35]; secondly, it may be the same, as most boys’ food intake is much higher than that of girls, and boys have more of a preference for meat and potato food, which is related to overweight and obesity [36]; thirdly, in terms of life behavior, the 2005 Adolescent Risk Behavior Monitoring Report pointed out that 29.1% of boys play computer games for more than 2 h a day, which twice as much as girls play [37].

Many scholars have explored the distribution characteristics of overweight and obesity in children and adolescents in China, and the results of the study have shown that the prevalence of overweight and obesity is higher in the northern region than in the western region, and the central and southern regions are in the middle [12,38]. The reason for this phenomenon may be the result of the interaction of natural and social environmental factors. Heilongjiang is located in northeast China, with high latitude and cold climate. Its special climatic conditions restrict outdoor activities of children and adolescents and require high-energy food to supplement the body’s calories [39,40]. Shanghai is located in eastern China. As an economically developed coastal city, children and adolescents exhibit more sedentary types of behavior and a more complete dietary structure and are more likely to be exposed to a rich food environment and a more extensive obesity environment [41,42]. However, places such as Guizhou and Yunnan have higher elevations. Due to special natural conditions, inconvenient transportation, and a relatively underdeveloped economy in high-altitude areas, children and adolescents cannot obtain adequate nutritional supplements during their growth and developmental stages [43].

### 4.2. Analysis of the Relationship between Overweight and Obesity in Children and Adolescents and Factors Affecting the Natural Environment

The results of this study show that the prevalence of overweight and obesity in children and adolescents increases with the increase of latitude. The reasons may be as follows: First, in terms of behavioral factors, cold weather in high latitudes will restrict travel, causing people to reduce their exercise volume and slow down their metabolism; the climate conditions in low latitudes are pleasant, which can increase children and adolescents’ travel and metabolism. The enthusiasm for exercise of children and adolescents accelerates their metabolism and ultimately leads to a higher prevalence of overweight and obesity in children and adolescents in high latitudes compared to low latitudes [40]. Second, in terms of dietary structure, because of the different climatic conditions at different latitudes, the types of crops planted are also different. For example, the northern high latitudes are restricted by cold climate conditions, and there are fewer types of crops. Children and adolescents mainly rely on high-fat and high-cholesterol foods to be able to supplement the calories needed by the body over time [39]. However, these high-fat, high-cholesterol foods are more likely to cause fat accumulation. In the low latitudes of the south, due to the suitable temperature and the variety of crops, children and adolescents eat mostly foods such as potatoes, rice, and fish [44], foods that do not easily cause fat accumulation.

This study found that the prevalence of overweight and obesity in children and adolescents is lowest at high altitudes. The appearance of this situation may be related to the partial pressure of oxygen in the plateau area. The hypoxia caused by the cold temperature and low-pressure atmospheric environment in the plateau area is the most important decisive factor affecting the physical development of individuals in high-altitude areas [45]. The higher the altitude, the lower the partial pressure of oxygen. This low-oxygen environment can indirectly affect the development of other organs and skeletal muscles of children and adolescents by affecting the function of the exhalation system. Moreover, the higher altitude, the greater distance from the ocean, and the lack of trace elements (such as iodine) required by the human body in the air make the physical development of children and adolescents in high-altitude areas slower than that in low-altitude areas [14]. At the same time, high-altitude areas have inconvenient transportation due to harsh natural conditions. Many materials need to be transported and supplied from other places, and economic development is relatively underdeveloped compared with low-altitude areas. An underdeveloped economy will lower people’s material living standards, which will directly affect the growth and development of children and adolescents without adequate nutritional supplements over a long time, and malnutrition still exists. In addition, due to the special geographical environment, a high-altitude area has a single dietary structure and a year-round lack of fruits and vegetables. The trace elements obtained in the food are not enough to maintain the normal physical development of children and adolescents, which seriously affects the physical development of children and adolescents in the plateau area.

In summary, overweight and obesity of children and adolescents are affected by the two natural environmental factors of latitude and altitude. The main manifestation is that the risk of overweight and obesity in children and adolescents increases with the increase in latitude, while high altitude reduces the risk of overweight and obesity in children and adolescents.

### 4.3. Analysis of the Relationship between Overweight and Obesity in Children and Adolescents and Factors Affecting the Social Environment

Research results show that the detection rate of overweight and obesity among children and adolescents in China increases with the increase in SES levels. Overweight and obesity in children and adolescents in developing countries such as India and Nigeria also occur in families with higher socioeconomic status [46]. The main reason for this phenomenon is that with the rapid development of China’s economy and an abundant food supply, people’s lifestyles and dietary patterns have undergone great changes [47], but parents’ nutritional knowledge and awareness of balanced diets have not kept pace with economic development [48]. 

The annual growth value of China’s urbanization increased from 0.53% in 1991 to 1.61% in 2010 [49], which is consistent with the trend in the prevalence of overweight and obesity among children and adolescents. This may be because the higher the urbanization is, the easier it is to meet the food requirements of children. The weekly intake of fish, meat, soy products, dairy products, fruits, vegetables, etc., is significantly higher in urban areas than in rural areas, which leads to far more overweight and obese children in cities than in rural areas [50]. Secondly, due to changes in lifestyles, urban children and adolescents have reduced physical activity time while increasing static behaviors [51]. Finally, air pollution is also a potential factor that makes urban children and adolescents more overweight and obese than those in rural areas. Studies have shown that exposure to high concentrations of air pollution is positively correlated with overweight and obesity in children aged 2–13 years [52]. However, it is worth noting that the low rate of overweight and obesity among rural children and adolescents does not mean that there is no need to pay attention to this issue. A study by Zhang et al. pointed out that between 1985 and 2014, the prevalence of overweight and obesity among rural children in Shandong province rose rapidly, and the problem of overweight and obesity in rural areas should not be ignored [53].

The research results show that the higher the GDP, the greater the risk of overweight among children and adolescents. The prevalence of overweight in children and adolescents in upper-middle-income and high-income regions is much higher than that of children and adolescents in low-middle-income regions. There is a certain correlation and coordination between China’s per capita GDP and urbanization rate. The higher the per capita GDP, the higher the level of urbanization, and the lower the per capita GDP, the lower the level of urbanization [54]. Therefore, the trend in the prevalence of overweight and obesity among children and adolescents with different incomes is consistent with the results under the degree of urbanization. The reason why the risk of overweight and obesity in children and adolescents increases with the increase in GDP may be that a higher GDP level makes it easier for children and adolescents to be exposed to a rich food environment and a more extensive obesity environment, which leads to overweight and obesity rates in children and adolescents higher than children and adolescents at low GDP levels [41]. 

In summary, the social environmental factors of overweight and obesity in children and adolescents are family SES level, GDP, and degree of urbanization. The main manifestations are as follows: the risk of overweight in children and adolescents increases with the increase in family SES levels and GDP; the higher the degree of urbanization, the greater the risk of obesity in children and adolescents.

The advantage of this study lies in the comparative analysis of the BMI of children and adolescents at different altitudes and latitudes through a large-scale survey to understand the distribution characteristics and prevalence of overweight and obesity in children and adolescents aged 10 to 18 in China. At the same time, in terms of natural environmental factors and social environmental factors, the impact of environmental factors such as latitude, altitude, family SES, and urbanization degree on children and adolescents’ overweight and obesity is discussed. This is designed to improve the physical and healthy development of children and adolescents and to develop targeted control in children. Individualized interventions for overweight and obesity in adolescents provide relevant theoretical basis. Against the background of the increasing global nutritional status of overweight and obesity among children and adolescents, countries should combine their own natural and social environmental factors to carry out interventions to control overweight and obesity in children and adolescents to curb the increasing trend of overweight and obesity.

The disadvantage is that, due to the limited cognitive abilities of children and adolescents aged 7–9, the family situation component of this study cannot be completed independently. It is hoped that, in subsequent studies, with the help of teachers and parents, the status of overweight and obesity in children and adolescents of all ages and its influencing factors will be analyzed.

## 5. Conclusions

This study took a sample of 26,120 Chinese children and adolescents aged from 10 to 18. Through an analysis of the overweight and obesity status of children and adolescents and their environmental factors, the following conclusions were drawn:

(1) Among children and adolescents aged 10 to 18, the prevalence of overweight and obesity generally decreases with age, and the overweight and obesity rates of boys are higher than those of girls. The highest was at the age of 10, but the overweight prevalence was highest at the age of 11.
(2) The risk of overweight and obesity in children and adolescents increases with latitude; high altitude reduces the risk of overweight and obesity in children and adolescents.
(3) The risk of overweight in children and adolescents increases with the increase in family SES and GDP; the higher the degree of urbanization, the greater the risk of obesity in children and adolescents.

## Figures and Tables

**Table 1:** Basic characteristics of the research objects.

	Boy (*n* = 13,068)		Girl (*n* = 13,052)		Total (*n* = 26,120)	
	*N*	Ratio/%	*N*	Ratio/%	*N*	Ratio/%
Latitude						
Low	4961	38	4966	38	9927	38
Middle	5576	42.7	5631	43.1	11,207	42.9
High	2531	19.4	2455	18.8	4986	19.1
Altitude						
Low	9154	70	9201	70.5	18,355	70.3
Medium	2471	18.9	2330	17.9	4801	18.4
High	1443	11	1521	11.7	2964	11.3
SES						
Low	1640	12.5	1862	14.3	3502	13.4
Middle	5682	43.5	5827	44.6	11,509	44.1
High	5746	44	5363	41.1	11,109	42.5
Degree of urbanization						
Worst	1003	7.7	1229	9.4	2232	8.5
Lower	3003	23	3057	23.4	6060	23.2
Middle and upper	2511	19.2	2439	18.7	4950	19
Best	6551	50.1	6327	48.5	12,878	49.3
GDP						
Medium income	788	6	766	5.9	1554	5.9
Middle and upper	8933	68.4	9043	69.3	17,976	68.8
High income	3347	25.6	3243	24.8	6590	25.2
Engel coefficient						
Well-off	992	7.6	979	7.5	1971	7.5
Rich	6549	50.1	6525	50	13,074	50.1
Richest	5527	42.3	5548	42.5	11,075	42.4

**Table 2:** Prevalence of different nutritional status in children and adolescents aged 10–18 years (%).

Age (Years)	*N*	Boys				*N*	Girls				Total	Total			
		Thinness	Normal Weight	Overweight	Obesity		Thinness	Normal Weight	Overweight	Obesity		Thinness	Normal Weight	Overweight	Obesity
10	1437	5.2	54.5	22.6	17.7	1482	6.2	71.7	15.8	6.3	2919	5.7	63.2	19.2	11.9
11	1509	4.8	56.3	23.3	15.6	1476	7.0	70.0	17.8	5.2	2985	5.9	63.1	20.6	10.5
12	1473	5.4	61.4	21.9	11.3	1469	8.0	75.0	12.4	4.6	2942	6.7	68.2	17.1	7.9
13	1550	6.6	66.0	18.1	9.3	1510	5.4	79.5	11.5	3.6	3060	6.0	72.6	14.8	6.5
14	1544	7.6	71.7	15.1	5.6	1497	3.9	84.9	8.7	2.5	3041	5.8	78.2	11.9	4.0
15	1541	5.1	73.6	15.6	5.7	1500	4.1	86.1	8.5	1.3	3041	4.6	79.8	12.1	3.6
16	1473	6.4	74.1	13.4	6.0	1534	4.6	87.0	7.1	1.4	3007	5.5	80.7	10.2	3.6
17	1379	6.8	75.1	13.5	4.6	1382	3.3	90.4	5.5	0.8	2761	5.0	82.8	9.5	2.7
18	1162	6.4	80.0	10.1	3.5	1202	3.9	90.2	4.7	1.2	2364	5.1	85.2	7.4	2.3
**Tatal**	13,068	6.0	67.8	17.2	8.9	13,052	5.2	81.4	10.4	3.0	26,120	5.6	74.6	13.8	6.0

**Table 3:** Prevalence of nutritional status of children and adolescents in different provinces (%).

Province	*N*	Boy				*N*	Girl				Total	Total			
		Thinness	Normal Weight	Overweight	Obesity		Thinness	Normal Weight	Overweight	Obesity		Thinness	Normal Weight	Overweight	Obesity
Shanghai	900	2.6	64.1	21.8	11.6	880	2.6	80.0	13.6	3.8	1780	2.6	72	17.8	7.7
Heilongjiang	842	7.1	58.3	20.7	13.9	779	5.4	75.7	13.9	5.0	1621	6.3	66.7	17.4	9.6
Hebei	724	7.0	65.1	18.2	9.7	865	4.9	80.8	11.4	2.9	1589	5.9	73.6	14.5	6
Henan	835	5.4	70.2	17.0	7.4	792	5.4	83.3	8.0	3.3	1627	5.4	76.6	12.6	5.4
Shanxi	819	4.6	66.2	19.0	10.1	768	3.8	80.5	11.7	4.0	1587	4.2	73.1	15.5	7.2
Jiangsu	778	3.5	72.8	17.6	6.2	767	3.3	85.5	8.6	2.6	1545	3.4	79.1	13.1	4.4
Zhejiang	900	6.6	69.4	16.0	8.0	900	6.0	80.9	10.2	2.9	1800	6.3	75.2	13.1	5.4
Anhui	738	6.9	69.5	15.7	7.9	733	9.1	79.8	8.3	2.7	1471	8	74.6	12	5.3
Jiangxi	880	9.0	75.6	10.6	4.9	900	9.8	84.0	4.2	2.0	1780	9.4	79.8	7.4	3.4
Sichuan	727	6.1	69.9	14.6	9.5	707	2.7	83.2	12.2	2.0	1434	4.4	76.4	13.4	5.8
Guizhou	724	6.2	74.3	13.0	6.5	807	5.0	86.9	6.7	1.5	1531	5.6	80.9	9.7	3.9
Fujian	791	4.7	61.6	23.5	10.2	775	3.6	81.5	11.1	3.7	1566	4.2	71.5	17.4	7
Hainan	900	8.7	70.8	13.3	7.2	900	5.8	83.1	8.3	2.8	1800	7.2	76.9	10.8	5
Xinjiang	788	3.8	65.1	20.2	10.9	775	4.1	80.5	13.2	2.2	1563	4	72.7	16.7	6.6
Jilin	900	9.3	59.8	19.0	11.9	900	7.3	75.2	13.1	4.3	1800	8.3	67.5	16.1	8.1
Yunnan	822	4.7	73.4	15.5	6.4	804	3.5	82.5	11.6	2.5	1626	4.1	77.9	13.5	4.5

**Table 4:** Prevalence of overweight and obesity in children and adolescents under different natural environmental factors (%).

Items	Level	Boys			χ^2^	*p*	Girls			χ^2^	*p*	Total			χ^2^	*p*
		Thinness	Overweight	Obesity			Thinness	Overweight	Obesity			Thinness	Overweight	Obesity		
Latitude					100.136	0.000				61.290	0.000				157.652	0.000
	Low	6.6	15.5	7.3			5.8	8.8	2.6			6.2	12.1	4.9		
	Middle	5.2	17.6	8.8			4.4	10.4	3.1			4.8	14	5.9		
	High	6.9	19.9	12.2			5.7	13.4	3.9			6.3	16.7	8.1		
Altitude					56.307	0.000				49.952	0.000				99.785	0.000
	Low	6.4	17.4	8.9			5.7	10	3.2			6	13.7	6		
	Medium	5.1	18.7	10.8			3.7	13	3.1			4.4	15.9	7.1		
	High	5.7	13.8	5.9			4.3	8.7	1.8			5	11.2	3.8		

**Table 5:** Prevalence of overweight and obesity in children and adolescents under different socioeconomic conditions (%).

Items	Grade	Boys			χ^2^	*p*	Girls			χ^2^	*p*	Total			χ^2^	*p*
		Thinness	Overweight	Obesity			Thinness	Overweight	Obesity			Thinness	Overweight	Obesity		
SES					133.566	0.000				52.566	0.000				178.592	0.000
	Low	8	11.2	5.8			5.2	7.4	1.9			6.5	9.1	3.7		
	Middle	6.5	16.4	8.5			4.8	9.8	3.4			5.6	13	5.9		
	High	5	19.8	10.3			5.6	12	3			5.3	16.1	6.8		
Degree of urbanization					31.001	0.000				35.200	0.000				60.137	0.000
	Worst	7.8	15.6	8			4.8	9	2.1			6.1	12	4.7		
	Lower	6.8	16.6	9.9			6.4	10.6	3.1			6.6	13.6	6.4		
	Middle and upper	6.9	16.8	8.4			6.2	9.8	3.4			6.5	13.4	6		
	Best	5.1	18	8.8			4.3	10.7	3			4.7	14.4	6		
GDP					61.548	0.000				23.485	0.001				64.581	0.000
	Medium income	9.6	11.2	5.5			5.2	5.9	2.7			7.5	8.6	4.1		
	Middle and upper	6	17	9.3			5.4	10.9	3.1			5.7	13.9	6.2		
	High income	5.3	19.2	8.7			4.7	9.9	2.9			5	14.6	5.8		
Engel coefficient					68.703	0.000				36.859	0.000				78.199	0.000
	Well-off	9.3	11.1	5.5			4.8	6.5	2.5			7.1	8.8	4		
	Rich	5.4	17.4	9			5.7	10	2.7			5.6	13.7	5.9		
	Richest	6.2	18.1	9.4			4.7	11.4	3.5			5.4	14.8	6.4		

**Table 6:** Logistic regression of the influential factors of overweight in children and adolescents.

		OR (95%CI)	*p*
Gender			
	Boys	1.00	
	Girls	0.55 (0.52–0.60)	0.000
Latitude			
	Low	1.00	
	Middle	0.99 (0.87–1.12)	0.837
	High	1.33 (1.15–1.54)	0.000
Altitude			
	Low	1.00	
	Middle	0.99 (0.88–1.12)	0.853
	High	0.79 (0.66–0.95)	0.012
SES			
	Low	1.00	
	Middle	1.17 (1.03–1.34)	0.020
	High	1.41 (1.24–1.61)	0.000
Degree of urbanization			
	Worst	1.00	
	Lower	0.98 (0.83–1.16)	0.792
	Middle and upper	0.99 (0.83–1.18)	0.879
	Best	1.12 (0.96–1.32)	0.149
GDP			
	Medium income	1.00	
	Middle and upper	1.50 (1.02–2.21)	0.041
	High income	1.66 (1.11–2.47)	0.013
Engel coefficient			
	Well-off	1.00	
	Rich	0.91 (0.64–1.28)	0.579
	Richest	1.00 (0.70–1.44)	0.982

**Table 7:** Logistic regression of the influential factors of obesity in children and adolescents.

		OR (95%CI)	*p*
Gender			
	Boys	1.00	
	Girls	0.31 (0.28–0.35)	0.000
Latitude			
	Low	1.00	
	Middle	0.98 (0.81–1.17)	0.779
	High	1.52 (1.24–1.86)	0.000
Altitude			
	Low	1.00	
	Middle	0.85 (0.71–1.02)	0.072
	High	0.60 (0.45–0.80)	0.000
SES			
	Low	1.00	
	Middle	1.13 (0.93–1.38)	0.216
	High	1.18 (0.97–1.45)	0.099
Degree of urbanization			
	Worst	1.00	
	Lower	1.11 (0.86–1.43)	0.425
	Middle and upper	1.01 (0.77–1.32)	0.950
	Best	1.29 (1.01–1.65)	0.038
GDP			
	Medium income	1.00	
	Middle and upper	1.30 (0.71–2.37)	0.392
	High income	1.30 (0.70–2.40)	0.408
Engel coefficient			
	Well-off	1.00	
	Rich	0.87 (0.50–1.50)	0.614
	Richest	1.04 (0.59–1.82)	0.906

## Data Availability

The datasets generated and/or analyzed during the current study are not publicly available but are available from the corresponding author on reasonable request.
